# 978 Lessons Learned: Independent Utilization of the Burn Care Quality Platform (BCQP)

**DOI:** 10.1093/jbcr/iraf019.509

**Published:** 2025-04-01

**Authors:** Sarah Shpil, Kara Paulk, Lee Ann Wurster, Renata Fabia, Rajan Thakkar, Dana Schwartz

**Affiliations:** Burn Center at Nationwide Children’s Hospital; Nationwide Children’s Hospital; Nationwide Children’s Hospital; Nationwide Children’s Hospital; Nationwide Children’s Hospital; Nationwide Children’s Hospital

## Abstract

**Introduction:**

The Burn Care Quality Platform (BCQP) is a specialized data source designed to enhance tracking and management of burn patient data within healthcare organizations. This platform provides a structured approach to collecting and analyzing data related to burn injuries, interventions, and outcomes. Our institutional goal was to participate in the BCQP full platform to become independent of our previously used trauma registry and also enhance its overall ease of use.

**Methods:**

To support this transition, a Burn Data Coordinator position was created in April 2023, dedicating a 0.5 full-time equivalent to patient tracking and data entry. Onboarding resources included recorded webinars, virtual troubleshooting, and a training seminar at the American Burn Association (ABA) annual meeting. To streamline data requests, our Burn Data Coordinator created a dictionary that was designed to supplement the BCQP, enhancing overall data usability and ease of understanding by external users.

**Results:**

Within 13 months month of implementation, we achieved full independence from our previously used Trauma Registry. Since operationalizing, the BCQP has helped identify several performance improvement opportunities, leading to initiatives in the outpatient setting. The new requirement to track long term outcomes led to the revision of scar measurement documentation and of the providers’ burn clinic note template with data-enabled fields for more reliable abstraction into the BCQP. It also prompted the development of an interdisciplinary work group to create defined intervals for patient follow-up and streamline documentation of comparable data.

**Conclusions:**

Implementation of the BCQP has led to a pivotal shift in how we manage burn data within our organization. The establishment of the Burn Data Coordinator role and the use of various training resources have been crucial in operationalizing this new process. Transitioning to the BCQP full platform use has highlighted the need for an adjunct data dictionary. It has also been the catalyst for several quality/performance improvement initiatives within our pediatric burn center.

**Applicability of Research to Practice:**

Our experience with the BCQP provides valuable insights for other centers considering its adoption. It demonstrates the significant benefits of enhanced patient tracking and the platform’s capability to identify areas needing enhancement. Our vision as early adopters is to work with the ABA and other like centers to optimize use of the BCQP to generate quality data capture, improve functionality, and work toward a shared goal of improving burn patient outcomes.

**Funding for the Study:**

N/A

